# Identification of Genetic Variants Using Next-Generation Sequencing in Pediatric Myelodysplastic Syndrome: From Disease Biology to Clinical Applications

**DOI:** 10.3390/ijms26146907

**Published:** 2025-07-18

**Authors:** Viviane Lamim Lovatel, Gerson Moura Ferreira, Beatriz Ferreira da Silva, Rayane de Souza Torres, Rita de Cássia Barbosa da Silva Tavares, Ana Paula Silva Bueno, Eliana Abdelhay, Teresa de Souza Fernandez

**Affiliations:** 1 Cytogenetic Laboratory, Cell and Gene Therapy Program, Instituto Nacional de Câncer (INCA), Rio de Janeiro 20230-130, RJ, Brazil; viviane.lovatel@inca.gov.br (V.L.L.); beaferreira.ds@gmail.com (B.F.d.S.); rayanetorresbmd@gmail.com (R.d.S.T.); 2 Stem Cell Laboratory, Instituto Nacional de Câncer, Rio de Janeiro 20230-130, RJ, Brazil; gmferreirag@gmail.com (G.M.F.); eabdelhay@inca.gov.br (E.A.); 3 Bone Marrow Transplantation Center, Instituto Nacional de Câncer (INCA), Rio de Janeiro 20230-130, RJ, Brazil; ritacbt@gmail.com; 4 Faculdade de Medicina, Instituto de Pediatria e Puericultura Martagão Gesteira, Universidade Federal do Rio de Janeiro, Rio de Janeiro 21941-912, RJ, Brazil; apbueno65@gmail.com

**Keywords:** pediatric myelodysplastic syndrome, genetic variants, targeted NGS panel

## Abstract

This study aimed to identify genetic variants using a customized next-generation sequencing (NGS) panel for pediatric myelodysplastic syndrome (pMDS) and to explore their associations with cytogenetic and clinical characteristics. Cytogenetic analyses were conducted using G-banding and fluorescence in situ hybridization. NGS was performed with the Ion Torrent Personal Genome Machine for the following genes: *GATA2*, *RUNX1*, *CEBPA*, *ANKRD26*, *ETV6*, *SAMD9*, *SAMD9L*, *PTPN11*, *NRAS*, *SETBP1*, *DDX41*, *TP53*, *FLT3*, *SRP72*, and *JAK3*. Analyses were performed with Ion Reporter 5.20.8.0 software. Genetic variants were classified using the dbSNP, 1000 Genomes, COSMIC, and Varsome databases. We analyzed 25 cases of pMDS; 15 presented abnormal karyotypes, and 19 showed genetic variants. Among the 29 variants identified across 12/15 genes, 27% were pathogenic and 14% were likely pathogenic, with *NRAS* and *GATA2* most frequently associated with disease progression. A new somatic variant of uncertain significance in *SETBP1* was detected in seven patients showing heterogeneous clinical outcomes. Genetic variants were found in 7/10 patients with normal karyotypes, indicating that submicroscopic alterations can shed light on disease biology. Our results highlight the critical role of a targeted NGS panel in identifying molecular alterations associated with pMDS pathogenesis, thereby enhancing diagnostic precision, prognosis, and aiding in treatment selection.

## 1. Introduction

Pediatric myelodysplastic syndrome (pMDS) is a rare clonal hematopoietic disease that accounts for 5–10% of pediatric hematological malignancies. This neoplasm is characterized by ineffective hematopoiesis and bone marrow dysplasia of one or more cell lines. It also carries a high risk of progression to acute myeloid leukemia (AML) [1,2]. Furthermore, diagnosing pMDS is particularly challenging due to the significant heterogeneity in clinical presentation. Its overlapping symptoms with other conditions, such as inherited bone marrow failure syndromes, also make the diagnosis more difficult [3].

In this context, cytogenetics has been an important tool for the diagnosis and risk stratification of MDS. Specific cytogenetic abnormalities, such as chromosome 7 alterations, are especially relevant in pediatric cases, holding significant prognostic value and guiding clinical management [4]. However, approximately half of the pMDS patients have normal karyotypes, underscoring the importance of identifying and integrating molecular data to advance our understanding of pMDS biology [1,4,5].

Recent advances in the genomic understanding of MDS have enhanced our knowledge of its molecular landscape. These insights have led to updates in the classification systems and the development of more refined prognostic models, thereby improving both diagnostic accuracy and risk stratification [2,3]. However, most of the knowledge about molecular alterations in MDS has been acquired from studies in adult patients [4,5]. This highlights a particular challenge, as pediatric cases of MDS often display significant differences, including variations in genetic profiles and treatment approaches, compared to adults [6].

The largest study to date that performed comprehensive whole-exome sequencing in a pMDS cohort analyzed 46 primary cases to better understand the somatic and germline variants associated with this neoplasm [5]. This study, along with previous research, revealed that in pMDS, unlike in adults, mutations in genes vital for epigenetic regulation, such as *TET2*, *ASXL1*, and *DNMT3A*, as well as RNA-splicing genes like *SF3B1* and *U2AF1*, are rare. In contrast, *Ras/MAPK* signaling pathway mutations are notably more prevalent in pediatric cases [5,6,7,8].

Pediatric MDS also shows a higher frequency of germline variants. Specifically, genetic variants in *GATA2* and *SAMD9/SAMD9L* are found in 7% and 8%, respectively, of pMDS patients with chromosome 7 alterations. Overall, *GATA2* is the most common genetic alteration associated with an increased risk of developing MDS and AML [8]. Moreover, germline mutations in other important genes, such as *RUNX1*, *ETV6*, *CEBPA*, *ANKRD26*, and *SRP72* have been detected in a small subset of pMDS patients [8,9,10]. Additionally, the precise mechanisms through which germline predisposition contributes to pMDS remain poorly elucidated. However, some studies have shown that the acquisition of somatic variants, depending on the underlying germline alteration, may contribute to either increased disease aggressiveness with progression to leukemia or hematopoietic stem cell adaptation, resulting in improved hematopoiesis [8,9]. Therefore, the evaluation and identification of these variants have implications for treatment strategies and prognostic results [11,12,13].

Due to the rarity of pMDS, studies evaluating the genomic architecture and clinical implications of these alterations in the pediatric age group are still limited. Moreover, the existing myeloid panels used in next-generation sequencing (NGS) were primarily developed based on the biology of MDS in adult patients [3]. Consequently, a thorough evaluation of genetic variants specific to pediatric cases is essential, as it may significantly influence risk stratification, therapeutic decisions, and prognostic assessment for this age group [11,12,13]. Therefore, the present study aimed to analyze genetic variants in pMDS using a customized NGS panel and investigate their association with clinical and cytogenetic characteristics, with a particular focus on the progression from MDS to acute myeloid leukemia (AML).

## 2. Results

### 2.1. Clinical and Cytogenetic Characteristics

This study was carried out on BM samples of twenty-five pediatric patients diagnosed with MDS, fifteen males and ten females. The median age at diagnosis was eight years (ranging from three months to seventeen years). Of the 25 patients, 84% (21/25) exhibited hypocellular bone marrow (BM). Pancytopenia, characterized by the involvement of all three cellular lineages, was present in 48% (12/25) of the cases. Among the patients, thirteen were diagnosed with refractory cytopenia of childhood (RCC) and twelve were classified into advanced subtypes, including eight cases of myelodysplastic syndrome with excess of blasts (MDS-EB) and three cases of MDS/AML. During the follow-up period, disease progression was observed in 56% (14/25) of patients (Table 1).

Abnormal karyotypes were observed in 60% (15/25) of the patients at diagnosis. Chromosome 7 alterations were present in five patients; among them, one had biclonal alteration (patient n.3), one had clonal evolution with the acquisition of del(X)(q23) (patient n.5), and three had monosomy 7 as the sole chromosomal abnormality. Besides this, during subsequent follow-ups with these patients, a patient with RCC and transitory monosomy 7 was observed (patient n.1). This patient was diagnosed at three months old and had a progressive loss of monosomy 7. At two years old, monosomy 7 could only be detected by fluorescence in situ hybridization (FISH) analysis in 7% of the BM cells, and one year later, this genetic alteration was no longer detectable. Complex karyotypes were detected in three patients with advanced pMDS subtypes and were associated with evolution from MDS to AML. Chromosomal translocations were present in three patients. These chromosomal alterations were associated with the evolution to AML. Trisomy 8, as the sole chromosomal abnormality, was observed in one patient (patient n.11), who showed evolution to AML. Interestingly, trisomy 8 was present in cases with chromosomal translocations (patients n.10 and n.25), complex karyotypes (patients n.7 and n.8), associated with del(11)(q23) (patient n.13), involved in clonal evolution associated with add(12)(p12) (patient n.12), and also present in a biclonal chromosomal abnormality (patient n.24). All these patients showed evolution from MDS to AML. Concerning the ten patients with normal karyotypes, only two were diagnosed with advanced subtypes, and only one showed evolution to AML (Table 1).

### 2.2. Genomic Alterations

Genetic variants were identified in 76% of cases (19/25), encompassing 12 different genes: *NRAS*, *GATA2*, *FLT3*, *ANKRD26*, *SRP72*, *SETBP1*, *SAMD9*, *SAMD9L*, *DDX41*, *JAK3*, *ETV6*, and *CEBPA*. In total, 29 variants were identified, with 27.6% (8/29) classified as pathogenic, 10.3% (3/29) as likely pathogenic, and 62.1% (18/29) as variants of uncertain significance (VUS). Concerning the consequences of the variants, missense variants represented 68.97% (20/29), followed by frameshift insertion at 13.8% (4/29) and frameshift deletion at 13.8% (4/29). Based on the variant allele frequency (VAF) of ≥ 40%, most pathogenic (5/8) and likely-pathogenic (2/3) variants had a suggestive germline origin (Table 2). The controls did not show genetic variants among the studied genes.

Genetic variants in *SETBP1* were the most frequent, corresponding to 27.5% (8/29) of the total variants identified in this study, followed by *NRAS* and *GATA2* with 13.79% (4/29) each. The genes with the highest number of pathogenic variants were *NRAS* and *GATA2*, though *FLT3* and *SETBP1* also had variants classified as pathogenic. Likely-pathogenic variants were also detected in *GATA2*, as well as in *ETV6* and *DDX41*.

We identified genetic variants that were not previously reported; they were detected in four genes: *SAMD9L* (c.2663C>T/p.Ser888Phe), *ETV6* (c.1359A>G/p.Ter453Trp), *GATA2* (c.-3CCGG>G), and *SETBP1* (c.3461A>C/p.His1154Pro). Notably, the novel variant in *SETBP1* was observed in seven patients. Among them, six cases involved the variant as an isolated finding. The allele frequency (VAF) of this novel variant ranged from 2.86% to 6.14%, with robust sequencing coverage exceeding 2000 reads.

Isolated genetic variants were identified in eleven patients, predominantly affecting the *SETBP1* (6/11) and *GATA2* (3/11) genes. However, only two cases with the *SETBP1* variant (patients n.19, 20) and one case with the *GATA2* variant (patient n.15) had no associated cytogenetic alteration. The other patients had abnormal karyotypes, including two complex karyotypes (patients n. 7, 8), one translocation (patient n.10), and one trisomy 8 (patient n.11) associated with the *SETBP1* variant. Meanwhile, the *GATA2* variant was associated with monosomy 7 in both cases (patients n. 2, 3).

**Figure 1:**
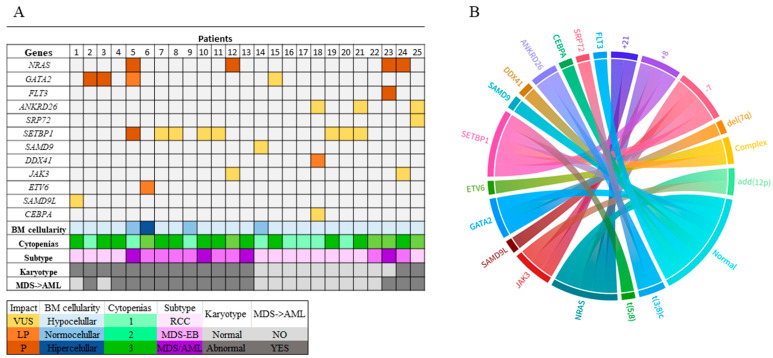
Overall clinical, cytogenetic, and genetic spectrum of pMDS patients. (**A**) The corresponding color coding is defined in the legend. P: pathogenic; LP: likely pathogenic; VUS: variant of uncertain significance; BM: bone marrow; RCC: refractory cytopenia of childhood; AML: acute myeloid leukemia; MDS-EB: myelodysplastic syndrome with excess of blasts. (**B**) The ribbon plot was created using Flourish [https://flourish.studio/ (accessed on 31 March 2025)], which shows the associations between cytogenetic profiles and genetic variants observed in the present study.

The association of genetic variants with clinical features showed that the patients with hypocellularity and pancytopenia also had chromosomal alterations and presented disease progression to AML. Concerning the clinical impact of genetic variants, the VUS were commonly observed in patients with RCC who lacked cytogenetic abnormalities and showed no disease progression. On the other hand, patients with EB-MDS and MDS/AML had a higher number of pathogenic and pathogenic-like variants. These patients also presented cytogenetic alterations, mainly involving chromosomes 7 and 8 (Figure 1A,B).

## 3. Discussion

In this study, a customized NGS panel for 15 genes was designed to analyze genetic variants in pMDS. These genes were selected based on a literature review focusing on their role in the pathogenesis of pMDS and genome sequencing in pMDS [5,8,10]. Based on previous studies, we used a VAF > 40% to indicate a germline origin of genetic variants [3,5]. In our study, 44.8% (13/29) of the variants were found to have a possible germline origin (Table 2). Our results reinforce the important role of germline susceptibility in pMDS [5,8].

Our results showed that higher frequencies of pathogenic variants were identified in *NRAS* and *GATA2*. Around 10–30% of myeloid malignancies have *NRAS* variants associated with an aggressive disease, presenting increased proliferation and resistance to therapies [15]. In MDS, there are three prevalent mutational hotspots: G12, G13, and Q61+ [15,16]. Here, we observed four patients with *NRAS* variants located within these hotspots. Two patients had the variant at G12 that is particularly prevalent in MDS, accounting for 50–70% of the cases with variants in this gene [5,15].

*NRAS* encodes a GTPase that, when mutated, constitutively binds to GTP, promoting oncogenesis. However, *NRAS* variants do not always function as independent prognostic factors. Their interaction with other genetic alterations is common and can lead to varying outcomes. For instance, *NRAS* mutations in combination with *TP53* have been associated with the development of lethal AML [15,16]. In our study, all patients with *NRAS* variants presented additional alterations. Patient n. 5 had *GATA2* and *SETBP1* gene variants; three exhibited chromosomal 7 alterations (patients n. 2, 3, and 5). These patients had disease evolution in accordance with the synergic adverse impact suggested for *NRAS* variants with additional alterations [15].

Regarding *GATA2*, three patients with monosomy 7 had pathogenetic or likely-pathogenic variants with a suggestive germline origin [5]. *GATA2* encodes a major hematopoietic transcription factor that plays an important role in early hematopoiesis. In pMDS, *GATA2* alterations are common genetic variants, representing 7% of all patients and 66.67% of those with chromosome 7 alterations, mainly in advanced subtypes [9,17]. In the present study, *GATA2* variants were observed in two patients with RCC and one with MDS/AML.

In pediatric patients with MDS, an association between monosomy 7 or del(7q) and genetic variants in *SAMD9/SAMD9L* has also been reported. However, unlike *GATA2*, it is predominantly observed in the initial stage of pMDS, the subtype RCC [9]. In the present study, *SAMD9L* and *SAMD9* variants were also observed only in patients with RCC. Interestingly, one of these patients had transient monosomy 7 (patient no. 1), and after approximately three years, the cytogenetic alteration was no longer detectable. This patient harbored two variants: a somatic one (p.Phe1307Ser) and a previously unreported germline variant in *SAMD9L* (p.Ser888Phe), located in the region that encodes a P-loop/NTPase protein, which plays a crucial role in several cellular processes, including apoptosis. Transient monosomy 7 has also been described in other studies, attributing this phenomenon to an adaptive response resulting from the somatic genetic rescue of variants in *SAMD9/SAMD9L* [5,9,18,19].

Likely-pathogenic variants were also identified in the *ETV6* and *DDX41* genes, both of which are key to hematopoiesis in myeloid neoplasms [2,3,20,21]. Here, patient no. 6, with the *ETV6* variant, exhibited a complex karyotype, a very poor prognosis, and disease relapse after HSCT. *ETV6* functions as a transcriptional repressor, playing a crucial role in maintaining hematopoiesis while also acting as a tumor suppressor [20,22]. Alterations in *ETV6* may interact with other genetic alterations to form a network that drives the self-renewal of leukemic stem cells and facilitates disease progression [20,22]. *DDX41* is vital for innate immunity, genomic stability, mRNA splicing, and ribosome biogenesis. Dysfunctional *DDX41* predisposes individuals to neoplasia, mainly of myeloid origin, although the pathogenic mechanisms are not fully understood [21]. In our study, patient no.18, who had a normal karyotype alongside *DDX41* variants, as well as variants in *ANKRD26* and *CEBPA*, responded well to HSCT.

In the present study, most of the variants detected were classified as VUS. This finding has become increasingly common in cancer research with the widespread adoption of massively parallel sequencing technologies. However, in hematologic malignancy, the limited available literature on VUS in genomic databases makes their interpretation challenging [23]. Recently, VUS in genes associated with myeloid neoplasms have been identified as markers of clonal hematopoiesis and potential indicators of prognosis in leukemia and myelodysplasia [24]. Therefore, reporting VUS and thoroughly assessing their potential impact is crucial for advancing the understanding of their role, especially in rare diseases where the genetic landscape is less well-characterized [22].

Among the genes in this panel, variants in *SETBP1* were observed in eight patients, being the most frequent VUS. It is important to note that our study identified a novel somatic variant, p.His1154Pro SETBP1, which was present in seven patients. Regarding these seven patients, six had only this genetic variant among the genes studied. This genetic variant is located at the AT-hook domain of transcription factor *SETBP1*, which is essential for DNA binding to promoter regions. Alterations in this domain lead to the upregulation of several genes involved in cellular processes important for hematopoiesis, such as *MECOM*, *HOXA9/10*, and *RUNX1* [25]. Beyond that, a previous study showed that a single *SETBP1* variant could be associated with a worse prognosis in myeloid neoplasms, regardless of the variant classification as VUS [22,25]. In our cases, the outcome of patients with this alteration was variable. Three patients with RCC showed normal karyotype and no disease progression. Conversely, in advanced subtypes, all patients had cytogenetic alterations mainly involving chromosomal gains and presented with disease evolution. These results showed a probable pathway involving the variant in *SETBP1* and an abnormal karyotype, which might involve trisomy 8. However, these results underscore the need for further research on the genomics of pMDS to understand the impact of this novel genetic variant in the biology of pMDS.

In this study, over half of the patients with normal karyotypes exhibited genetic variants. For example, one patient had a normal karyotype and two pathogenic variants, one in *NRAS* and one in *FLT3*. This patient presented an advanced subtype at diagnosis and an unfavorable outcome, with evolution to AML. Altogether, these results demonstrated that investigating submicroscopic alterations in patients with normal karyotypes can shed light on the molecular mechanisms involved in disease development and progression, as well as identify potential novel treatment targets. Nowadays, allogeneic hematopoietic stem cell transplantation remains the only potentially curative treatment for pMDS, and the rapid indication of this treatment is crucial for optimizing patient outcomes [14,26]. Therefore, identifying molecular biomarkers that may indicate rapid disease progression is essential to aid patient clinical management.

The genomic landscape of adult MDS has been extensively explored, allowing the development of commercial NGS panels. However, some essential genes in the pathology of pMDS are not included [2,3]. Despite some limitations of this study, such as the small number of patients analyzed, retrospective data collection, and the absence of skin fibroblast genomic analyses, this study showed the importance of using an NGS panel focused on pMDS molecular alterations. The use of this targeted panel with only 15 genes was able to bring new information about the biology of pMDS, allowing these results to be applied in the clinic. Such an approach enhances diagnostic and prognostic accuracy.

Currently, investigating molecular alterations through high-coverage sequencing platforms allows for the identification of new alterations that may have clinical relevance. Studies utilizing this approach open a new avenue for future research in pMDS, guiding treatment toward precision medicine.

## 4. Material and Methods

### 4.1. Patients and Controls

This study was conducted on retrospective BM samples sent to the cytogenetic laboratory from 2010 to 2023 as part of standard care for cytogenetic analyses and also as a research study, for which the remaining aliquots were used for molecular analyses. The patients were diagnosed at Instituto Nacional de Câncer (Rio de Janeiro, Brazil) and Instituto de Puericultura e Pediatria Martagão Gesteira (Rio de Janeiro, Brazil). The diagnosis was based on morphologic, cytogenetic, immunophenotypic, and clinical characteristics. None of the patients had previously been treated for malignancy, nor did they have a genetic syndrome. The subtype definition followed the International Consensus Classification of Myeloid Neoplasms [2]. We also used three bone marrow donor samples as controls for NGS analyses. This study was approved by the Ethics and Research Committee of the National Cancer Institute (reference number # 3401739) in accordance with the Declaration of Helsinki.

### 4.2. Cytogenetic Analyses

Classical cytogenetic analysis was performed on BM cells using G-banding [10]. Samples were initially collected in heparinized tubes. The cells were counted and cultured at a concentration of 5 × 10^6^ to 10 × 10^6^ cells/mL in a medium composed of 80% RPMI and 20% fetal bovine serum. The cultures were incubated at 37 °C in a 5% CO_2_ atmosphere for 24 h. To arrest cells in metaphase, colcemid was added to the cultures at a final concentration of 0.05 µg/mL during the final hour of incubation. After incubation, cells were subjected to hypotonic shock treatment using 0.075 M KCl for 20 min, and then fixed in a methanol/acetic acid solution (3:1 ratio). For the G-banding procedure, approximately 10 slides were prepared per patient. Fixed cells were dropped onto clean slides from a height and flame-fixed using a Bunsen burner. These slides were incubated at room temperature for about 24h to age before banding. The slides were incubated in a 0.1% trypsin solution in Dulbecco’s solution (8 g NaCl, 0.2 g KCl, 0.2 g KH_2_PO_4_, 1.5 g NaH_2_PO_4_ per 1 L distilled water, pH 7.8) for 1 s to 1 min. After incubation, they were washed in 0.9% NaCl and stained with 2% Giemsa in phosphate buffer (14.075 g NaH_2_PO_4_ per 1 L of distilled water, pH 6.8) for 15 min. A minimum of 20 metaphases were analyzed per BM sample from a patient. Chromosomes were identified and arranged according to the International System for Human Cytogenomic Nomenclature (ISCN), 2020 [27]. Karyotype images were captured using the Ikaros Karyotyping System (MetaSystems, Zeiss, Altlussheim, Germany).

Fluorescence in situ hybridization (FISH) analyses were performed to confirm chromosomal alterations, particularly in cases where clone sizes detected by G-banding were small. For these analyses, cytogenetic culture samples were dropped onto clean slides using a water bath method, allowing them to dry on a heated plate at 42 °C. The slides were then incubated in 2× SSC buffer (prepared from 20× SSC: 3.0 M NaCl and 0.3 M sodium citrate, pH 7.0) for 20 min at room temperature. Following incubation, the slides were incubated in an ethanol series—70%, 90%, and 100%—for 2 min each at room temperature. The probes were prepared and applied according to the manufacturer’s instructions. FISH was conducted using the following probes: del(7q)/-7 (D7S486 “spectrum orange”/CEP7 “spectrum green”) and +8 (LSI c-MYC 8q24, “spectrum orange”). The hybridization area was covered with a coverslip and sealed with rubber cement (Marabu, Tamm, Germany). Slides were incubated for 16 h in a hybridization chamber (Thermobrite, Leica, Richmond, VA, USA). After hybridization, the slides were washed in 0.4× SSC + 0.3% Tween at 73 °C for 2 min, followed by washing in 0.2× SSC + 0.1% Tween at room temperature for 1 min. The slides were then counterstained with DAPI. Analysis was performed using a fluorescence microscope (Olympus BX51, Miami, FL, USA), and images were captured using the ISIS imaging system (MetaSystems, Zeiss, Altlussheim, Germany).

### 4.3. Next-Generation Sequencing

Genetic variants were analyzed using next-generation sequencing (NGS) on the Ion Torrent platform. A customized panel was employed, targeting complete coding regions with 98.84% coverage, which included 25 bp intronic padding around each exon. The panel generated 259 amplicon sets, divided into two pools: Pool 1, containing 132 amplicons, and Pool 2, containing 127 amplicons. Sequencing was performed using the human reference genome (hg19, GRCh37). The standard DNA amplicon size was 375 base pairs, yielding a total of 47,355 base pairs sequenced. Additionally, a total of 15 genes associated with pMDS were selected based on the published scientific literature [4,5,6]. The genes investigated in this panel were *GATA2*, *RUNX1*, *CEBPA*, *ANKRD26*, *ETV6*, *SAMD9*, *SAMD9L*, *PTPN11*, *NRAS*, *SETBP1*, *DDX41*, *TP53*, *FLT3*, *SRP72*, and *JAK3*.

Genomic DNA (20 ng) quantified by the Qubit dsDNA HS Assay Kit (Thermo Fisher Scientific, Waltham, MA, USA) was used to prepare libraries for NGS using the Ion AmpliSeq Library Kit 2.0 (2 pools by sample). Barcoded adapters (Ion Xpress Barcode Adapters, Thermo Fisher Scientific, Waltham, MA, USA) were added to each sample. The purification was performed with AMPure XP purification (Beckman Coulter, Brea, CA, USA), and the quantification of the libraries was performed using the Ion Library TaqMan Quantification Kit (Thermo Fisher Scientific, Waltham, MA, USA) via real-time PCR. The Ion One Touch 2 instrument, using the Ion PGM Hi-Q-View OT2 kit, was used to conduct the emulsion PCR. Library enrichment was performed with ionic sphere particles (ISPs) on the Ion One Touch ES instrument (Thermo Fisher Scientific, Waltham, MA, USA). Libraries were loaded onto the Ion 316 v2 chip and sequenced on the Ion Torrent PGM sequencer (Thermo Fisher Scientific, Waltham, MA, USA). All steps were followed according to the manufacturer’s guidelines. Sequence alignment and analyses were performed using Ion Reporter software 5.20.8.0 [available online: https://ionreporter.thermofisher.com (accessed on 18 June 2025)]. The average base coverage depth exceeded 1000×, with over 95% of bases sequenced on target. Variant identification and visual confirmation were performed using the Integrative Genomics Viewer (IGV) tool.

Further analyses were performed using Ensembl Variant Effect Predictor (VEP) [https://www.ensembl.org/info/docs/tools/vep/index.html (accessed on 15 February 2025)], and genomic variants with a variant allelic frequency (VAF) of ≥2% were evaluated through the following databases: dbSNP [https://www.ncbi.nlm.nih.gov/snp/ (accessed on 20 February 2025)], 1000 Genomes [https://www.internationalgenome.org/ (accessed on 20 February 2025)], COSMIC [https://cancer.sanger.ac.uk/cosmic/ (accessed on 20 February 2025)], ClinVar [https://www.ncbi.nlm.nih.gov/clinvar/ (accessed on 20 February 2025)], and Varsome [https://varsome.com/ (accessed on 20 February 2025)]. Benign and likely-benign variants were excluded from the results. Pathogenic and likely-pathogenic variants were annotated with ClinVar and COSMIC, while unreported variants were based on in silico analysis obtained from tools in Varsome, as well as the variants classified as VUS.

## Figures and Tables

**Table 1:** Clinical–pathological characteristics of pediatric patients with MDS.

Patient (n.)	Age/ Gender	Karyotype	Subtype	BM Cellularity	Cytopenia	Blast Counts (%)	Evolution from MDS to AML	HSCT	Alive
1	3 months/F	45,XX,-7[24]/46,XX[2]	RCC	hypocellular	N, T, A	0%	NO	NO	YES
2	10/M	45,XY,-7[26]	RCC	hypocellular	T	4.5%	YES	YES	NO
3	1/M	46,XY,del(7)(q22q32)[5]/45,XY,-7[15]	RCC	hypocellular	N, T, A	2%	NO	YES	NO
4	15/M	45,XY,-7[24]	RCC	hypocellular	N, T, A	3.8%	YES	NO	NO
5	11/F	45,XX,-7[25]/45,X,del(X)(q23),-7[5]	MDS/AML	normocellular	N	20–30%	YES	NO	NO
6 *	3/M	49,XY,del(3)(q21),del(6)(q21),+der(6)del(6)(q21)	MDS-EB	hypercellular	N, T	16%	YES	YES	NO
		+8,+der(12)del(12)(p11)[21]							
7	6/F	58,XX,+X,+3,+5,+6,+8,+10,+11,+12,+13,+18,+20,+21[5]/	MDS-EB	hypocellular	N, T, A	16%	YES	NO	NO
		58,XX,idem,dup(1)(q21q31)[14]/46,XX[21]							
8	5/M	52,XY,+6,+8,+14,+16,+19,+22[3]/46,XY[19]	MDS-EB	hypocellular	N, T, A	5.8%	YES	NO	NO
9	12/F	46,XX,der(2)t(2;15)(q37;q21)[25]	MDS-EB	normocellular	T	16%	YES	NO	NO
10	5/M	46,XY,t(5;8)(q32;q22)[23]/46,XY[8]	MDS/AML	hypocellular	N, T, A	28%	YES	NO	NO
11	16/M	47,XY,+8[30]/46,XY[25]	MDS-EB	hypocellular	N, T, A	10%	YES	NO	NO
12	13/M	46,XY,add(12)(p12)[19]/47,XY,+8,add(12)(p12)[11]	MDS-EB	hypocellular	N, T	10%	YES	YES	YES
13	1/M	47,XY,+8,del(11)(q23)[9]/46,XY[13]	MDS/AML	hypocellular	N, T, A	22%	YES	NO	NO
14	17/M	46,XY[30]	RCC	normocellular	T	0.3%	NO	NO	YES
15	2/F	46,XX[30]	RCC	hypocellular	N, T, A	0.5%	NO	NO	YES
16	10/F	46,XX[21]	RCC	hypocellular	T	0.6%	NO	NO	YES
17	10/F	46,XX[20]	RCC	hypocellular	N	0%	NO	NO	YES
18	5/F	46,XX[30]	RCC	hypocellular	N	1%	NO	YES	YES
19	1/M	46,XY[25]	RCC	hypocellular	N, T, A	2%	NO	NO	YES
20	16/M	46,XY[30]	RCC	hypocellular	T	1.5%	NO	NO	YES
21	7/M	46,XY[35]	RCC	hypocellular	N, T, A	1%	NO	NO	YES
22	1/M	46,XY[20]	MDS-EB	hypocellular	N, T	18%	NO	NO	NO
23	16/M	46,XY[24]	MDS/AML	hypocellular	N, T	20%	YES	NO	NO
24	4/F	47,XX,+21[5]/47,XX,+8[3]/46,XX[20]	MDS-EB	hypocellular	N, T, A	15%	YES	NO	NO
25 *	4/F	46,XX,t(3;8)(q29;q11)c[26]	RCC	hypocellular	N, T	5.4%	YES	NO	YES

RCC: refractory cytopenia of childhood; MDS-EB: myelodysplastic syndrome with excess of blasts; AML: acute myeloid leukemia; N: neutropenia; T: thrombocytopenia; A: anemia. * Patients previously reported [12,14].

**Table 2:** Genetic variants identified in patients with pMDS.

Patient (n.)	Gene	Locus	dbsnp	Impact	Consequence	Genotype	VAF (%)	Protein	Coding
1	*SAMD9L*	chr7: 92761365	rs1199597457	VUS	M	A/G	10.76	p.Phe1307Ser	c.3920T>C
	*SAMD9L*	chr7: 92762622	-	VUS	M	G/A	47.80	p.Ser888Phe	c.2663C>T
2	*GATA2*	chr3: 128200723	rs387906634	p	M	C/T	45.73	p.Arg361His	c.1082G>A
3	*GATA2*	chr3: 128205858	rs1291114301	P	FI	TC/TC	100.00	p.Glu6GlyfsTer179	c.16_17insG
5	*NRAS*	chr1: 115258747	rs121913237	P	M	C/T	50.52	p.Gly12Asp, p.?	c.35G>A, c.*2043G>A
	*GATA2*	chr3: 128200776	rs1313081073	LP	FD	TCTGGCGGCCGA/T	64.71	p.Ser340LysfsTer40	c.1018_1028del TCGGCCGCCAG
	*SETBP1*	chr18: 42531907	rs267607042	P	M	G/A	48.95	p.Asp868Asn	c.2602G>A
6 *	*ETV6*	chr12: 12043980	-	LP	SP	A/G	3.32	p.Ter453Trp	c.1359A>G
7	*SETBP1*	chr18: 42532766		VUS	M	A/C	4.42	p.His1154Pro	c.3461A>C
8	*SETBP1*	chr18: 42532766	-	VUS	M	A/C	3.37	p.His1154Pro	c.3461A>C
10	*SETBP1*	chr18: 42532766	-	VUS	M	A/C	2.86	p.His1154Pro	c.3461A>C
11	*SETBP1*	chr18: 42532766		VUS	M	A/C	6.14	p.His1154Pro	c.3461A>C
12	*NRAS*	chr1: 115256530	rs1057519834, rs121913254	P	M	G/T	47.29	p.Gln61Lys	c.181C>A
	*JAK3*	chr19: 17953950	rs55778349	VUS	M	G/C	49.75	p.Pro151Arg	c.452C>G
14	*SAMD9*	chr7: 92733609	rs375515095	VUS	FD	ATT/A	52.69	p.Glu600AspfsTer12	c.1800_1801 delAA
15	*GATA2*	chr3: 128205877	-	VUS	FI	CCGG/C	4.05		c.-3CCGG>G
18	*DDX41*	chr5: 176943921	rs1554111842	LP	FI	T/TACCT	48.16	p.Arg10ValfsTer20	c.25_26 insAGGT
	*ANKRD26*	chr10: 27337805	rs1216270855, rs561705414	VUS	FD	CCAT/C	2.67	p.Asp579del	c.1736_173 delATG
	*CEBPA*	chr19: 33793023	rs1289919155	VUS	FD	CGCC/C	2.94	p.Gly104del	c.295_297delGGC
19	*SETBP1*	chr18: 42532766	-	VUS	M	A/C	3.62	p.His1154Pro	c.3461A>C
20	*SETBP1*	chr18: 42532766	-	VUS	M	A/C	3.21	p.His1154Pro	c.3461A>C
21	*ANKRD26*	chr10: 27389160	rs779596861	VUS	M	C/G	47.18	p.Glu32Asp	c.96G>C
	*SETBP1*	chr18: 42532766	-	VUS	M	A/C	4.51	p.His1154Pro	c.3461A>C
23	*NRAS*	chr1: 115258747	rs121913237	p	M	C/A	15.35	p.Gly12Val, p.?	c.35G>T, c.*2043G>T
	*FLT3*	chr13: 28609669	rs1568403015	P	FI	A/AG	8.79	p.Gly521TrpfsTer5	c.1559_1560insC
24	*NRAS*	chr1: 115258744	rs121434596	P	M	C/T	5.10	p.Gly13Asp, p.?	c.38G>A, c.*2046G>A
	*JAK3*	chr19: 17945970	rs1568403015	VUS	M	G/A	38.60	p.Arg657Trp	c.1969C>T
25 *	*SRP72*	chr4: 57361553	rs34419325	VUS	S	A/G	50.61	p.Lys557=	c.1671A>G
	*ANKRD26*	chr10: 27353007	rs12359281	VUS	M	ATC/GTC	50.40	p.Ile425Val	c.1273A>G

* Patients previously reported [12,14]; n.: patient number; P: pathogenic; LP: likely pathogenic; VUS: variant of uncertain significance; M: missense; FI: frameshift insertion; S: synonymous; FD: frameshift deletion; SP: stop loss; VAF: variant allelic frequency.

## Data Availability

The raw data supporting the conclusions of this article will be made available by the authors on request.
